# Supplementary material for: The Tyrosine Phosphatase Activity of PTPN22 Is Involved in T Cell Development via the Regulation of TCR Expression
Source: Int J Mol Sci. 2023 Sep 25;24(19):14505. doi: 10.3390/ijms241914505 (PMC10572452; doi:10.3390/ijms241914505)
Supplement: Supplementary file 1 [file ijms-24-14505-s001.zip › ijms-2600971-supplementary.pdf]

Figure S1

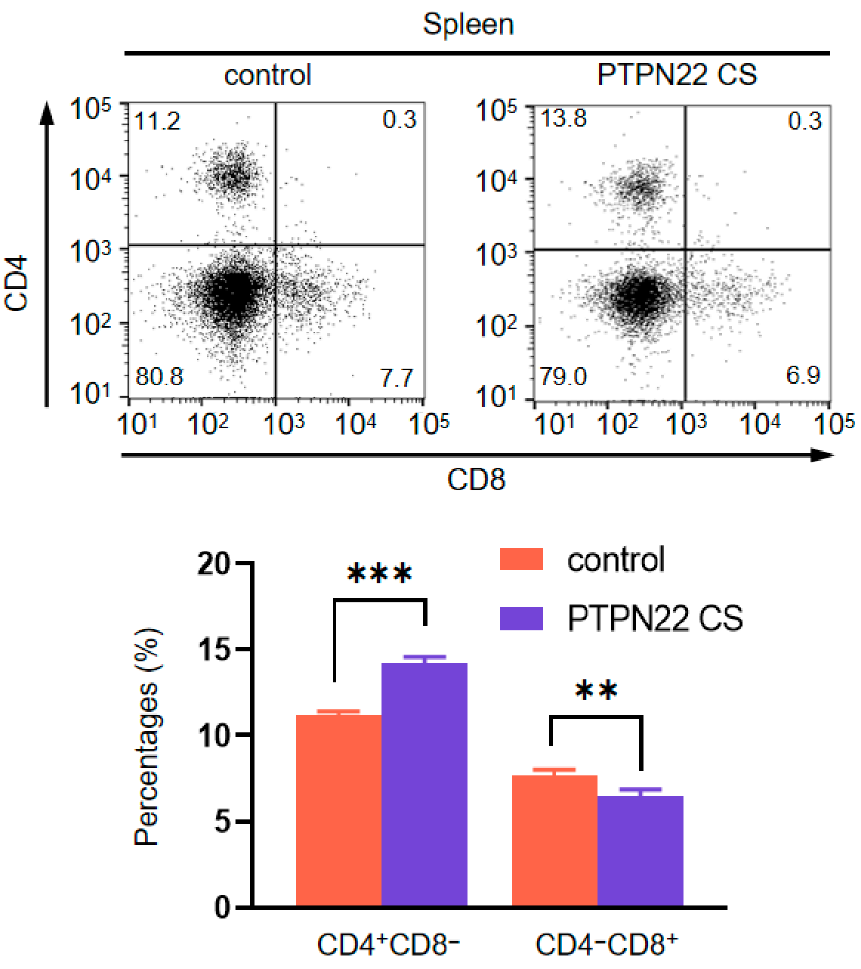

Figure S1. The proportion of CD4 cells in spleen of PTPN22 CS transgenic mice was increased. Taken the spleen cells from PTPN22 CS or control mice, washed them by Red Blood Cell Lysis Buffer. Detected the proportion of $CD4^-CD8^-$, $CD4^+CD8^+$, $CD4^-CD8^+$, and $CD4^+CD8^-$ cells in spleen. The experiments were repeated more than three times. The values were shown as mean ± SEM, n = 3. $^{**}p < 0.01$, $^{***}\ p < 0.001$.

Table S1 List of transgene-specific primers sequences for PCR

Primer	Sequence
Transgene PCR primer	F: 5'-TGATGTCTCCCAGGTAGTCC-3'<br>R: 5'-GGAATCCTCATCGGAAGTTA-3'
Internal control PCR primer	F: 5'-ACTCCAAGGCCACTTATCACC-3'<br>R: 5'-ATTGTTACCAACTGGGACGACA-3'<br>R: 5'-GACCTCAAACTTGGCAATACTCA-3'

Table S2 List of antibodies used in this study

Antibody	Cat#	Source	Dilution
PTPN22	14693	CST	1:1000
PLCγ1	5690	CST	1:1000
p-PLCγ1 (Y783)	2821	CST	1:1000
α-pErk1 (Thr202)/Erk2 (Tyr204)	04370	CST	1:1000
α-ZAP70	ab32429	Abcam	1:1000
α-pZAP70Tyr319	ab131270	Abcam	1:1000
α-Lck	ab3885	Abcam	1:1000
α-pLckTyr394	ab201567	Abcam	1:1000
α-Erk	ab17942	Abcam	1:1000
α-LAT	ab2507	Abcam	1:1000
α-pLAT (Tyr132)	ab4476	Abcam	1:1000
α-CD3ζ	ab226475	Abcam	1:1000
α-pCD3ζ (Tyr83)	ab68236	Abcam	1:1000
α-NFAT1	4389	CST	1:1000
α-IκB	9242	CST	1:1000
α-pIκB(Ser32)	2859	CST	1:1000
CD3ε	4443	CST	1:1000
Phospho-Tyrosine	9411	CST	1:1000

Fluor 488 Conjugated-IgG	4416	CST	1:500
β-actin	4970	CST	1:4000
α–GAPDH	2118	CST	1:4000
mouse CD3ε	16-0032-86	eBioscience.	1:1000
mouse CD28	16-0281-85	eBioscience.	1:2000
FITC-mouse-anti-TCRβ	562081	BD Pharmingen	1:300
PE-mouse-anti-CD3ε	561824	BD Pharmingen	1:500
APC-mouse-anti-CD3ε	553061	BD Pharmingen	1:500
PerCP-mouse-anti-CD4	561090	BD Pharmingen	1:300
APC-mouse-anti-CD4	561091	BD Pharmingen	1:500
FITC-mouse-anti-CD8	561966	BD Pharmingen	1:300
APC-mouse-anti-CD8	561093	BD Pharmingen	1:500
PE-mouse-anti-CD5	553025	BD Pharmingen	1:500
FITC-mouse-anti-CD25	561779	BD Pharmingen	1:300
PE-mouse-anti-CD44	561860	BD Pharmingen	1:300

Table S3 List of primer sequences for RT-PCR

Primer	Sequence
18s	F: 5'-AGTCCCTGCCCTTTGTACACA-3'<br>R: 5'-CGATCCGAGGGCCTCACTA-3'
Ptpn22	F: 5'-CAGCAACTACTGAAAGAAGCCC-3'<br>R: 5'-TAGGTCGCCTTCCTCCCTTCCAC-3'
IL-2	F: 5'-AGTGCCTAGAAGATGAACTTGGAC-3'<br>R: 5'-CATCTCCTCAGAAAGTCCACCAC-3'
IL-7	F: 5'-ATAGTAATTGCCCGAATAATGAACC-3'<br>R: 5'-GTGCCTTGTGATACTGTTAGTAAGTGG-3'
IFN-γ	F: 5'-ACTCAAGTGGCATAGATGTGGAA-3'<br>R: 5'-GACCTCAAACTTGGCAATACTCA-3'
